# Measurement of vaccination coverage at age 24 and 19–35 months: a case study of multiple imputation in public health

**DOI:** 10.1186/1478-7954-3-6

**Published:** 2005-07-05

**Authors:** Tammy A Santibanez, Lawrence E Barker, Kate M Shaw

**Affiliations:** 1 Centers for Disease Control and Prevention, National Immunization Program, Atlanta, Georgia, USA

## Abstract

**Aim:**

Childhood immunization coverage in the United States (U.S.) is often measured at age 24 months or, in the National Immunization Survey (NIS) at age of interview, which is between 19 and 35 months. This paper compares these standards.

**Methods:**

Data from the NIS is used to compare immunization coverage at time of interview, retrospectively among all children aged 24 or more months at time of interview, and obtained via multiple imputation (with 10 imputations) for all children, both nationally, by state, and by demographic groups.

**Results:**

At the national level, the difference between the 19–35 month estimate and the 24 month complete-case estimate was 1.9 percentage points. For most but not all states and subgroups, the 19–35 month estimate was higher than the 24 month complete-case estimate. The difference between vaccination coverage measured at 19–35 months and 24 months ranged from -2.3 to 7.5 percentage points among states. For three states, the difference between the 19–35 month and 24 month complete-case estimate was more than 6 percentage points, in twelve states there was a 4–6 percentage point difference, and in sixteen states a 2–4 percentage point difference. Conversely, five states had higher 24 month complete-case estimates than 19–35 month estimates.

**Conclusion:**

We found that the coverages at 19–35 and 24 months differ such that they would rarely be adequate surrogates for one another, particularly at a state level. Multiple imputation, which is easily implemented, increases precision of estimates of coverage at age 24 months.

## 1. Introduction

Multiple imputation is a well-established statistical practice [1-6]; however it is rarely used in health care surveys. For example, the National Health Interview Survey limits reports to complete case analyses [7]. Here, we demonstrate how multiple imputation can be applied to public health surveys, using data from the National Immunization Survey (NIS).

The NIS is the primary means by which immunization coverage is measured among pre-school aged children in the U.S. The NIS, conducted annually since 1995 by the Centers for Disease Control and Prevention (CDC), provides annual estimates of vaccination coverage among children aged 19–35 months for the nation and for each of the 50 states and 28 selected urban areas. Coverage is measured among children aged 19–35 months, as of the time of household interview. Each year the CDC publishes these NIS estimates of coverage at 19–35 months of age [8,9].

Vaccination coverage is measured by many others at 24 months of age. For example, the Health Plan Employer Data and Information Set (HEDIS^®^), of the National Committee for Quality Assurance, has a Childhood Immunization Status measure to estimate vaccination coverage among managed care enrollees who turned two years old during the measurement year [10,11]. Second, the Retrospective Surveys of School Enterers' Immunization Records measured coverage at 24 months of age[12,13]. In addition, many state, county, and local surveys assess childhood vaccination coverage at 24 months of age [14-18]. Finally, CDC also provides coverage estimates at 24 months (and other benchmark ages) from the NIS on its website ; these estimates include only those children in the NIS who were 24–35 months at the time of interview, thus representing a subset of children in the complete NIS. Other benchmark ages are at ages younger than 19 months, so all children are included in these analyses.

It is plausible that coverage measured at 19–35 months might be higher than that measured at 24 months; some children have up to 12 months extra to obtain vaccinations and some have up to 6 months less time to obtain vaccinations [19]. Is 19–35 month coverage higher than 24 month coverage? How different are 19–35 month and 24 month coverage measures, and is the difference uniform across states and demographic subgroups? Here, we compare vaccination coverage measured at 19–35 months with 24 month coverage, obtained both through complete case analysis and multiple imputation. Further, we demonstrate how multiple imputation could improve precision of estimates of 24 months immunization coverage.

## Methods

### Survey Design and Collection of Data

The NIS is conducted annually by CDC to obtain national, state, and selected urban-area estimates of vaccination coverage for the U.S. non-institutionalized population of children aged of 19–35 months. The NIS is a random-digit-dialing survey of households with age-eligible children followed by a mail survey of the eligible children's vaccination providers to obtain the children's vaccination information [20]. Interviews are conducted with the household member with best knowledge of the child's immunizations (a female guardian in about 85% of the cases). Analyses are restricted to children whose vaccination history was verified by their providers.

This study uses data from the 2001 and 2002 NIS. The 2001 NIS included children born February 1998 – June 2000 while the 2002 NIS included children born February 1999 – June 2001. Adequate provider data were obtained from 23,642 children in the 2001 NIS and 21,410 children in the 2002 NIS. A complex weighting methodology is used in the NIS to adjust estimates for household non-response, households with multiple phone lines, and vaccination history non-response. Details of NIS methodology have been published previously, and are not repeated here [20,21].

### Definitions

We evaluated coverage with the 4:3:1:3:3 series. Being 4:3:1:3:3 up-to-date (UTD) was defined as having: 4 or more doses of diphtheria and tetanus toxoids and pertussis vaccine (DTP), 3 or more doses of poliovirus vaccine, 1 or more dose of any measles-containing vaccine (MCV), 3 or more doses of *Haemophilus influenzae *type b vaccine (Hib), and 3 or more doses of hepatitis B vaccine.

Race/ethnicity of the child was respondent-reported during the household interview. The ratio of household income to the poverty level ratio was calculated based upon reported household income, number of persons in the household and official U.S. Census Bureau thresholds for poverty. Children were categorized into one of four groups: living "above poverty" for household income/poverty ratios ≥ 125%, living "near poverty" for ratios 100%-124%, "intermediate poverty" for ratios 50%-99%, and "severe poverty" if the household income/poverty ratio was less than 50%. This four-class categorization of poverty better reflects the impact of poverty on vaccination status than the simple above/below poverty level dichotomization [22]. For 2001 and 2002 respectively, 86.2 and 87.4 % of the respondents provided household income data. For the same years, of those providing household income data, useable provider information was obtained from 73.6 and 70.2 % of the respondents; adjustments for provider response propensity are included in the NIS weights [23].

### Three Measures of 4:3:1:3:3 Vaccination Coverage

#### 19–35 Month Coverage

All vaccinations received up to the day that the NIS interview was conducted are included in this measure. This is the usually quoted coverage [8,9].

#### 24 Month Complete-Case Coverage

For the 24 month complete-case measure, all vaccinations received up to and including the child's second birthday are included. This measure is calculated only among the children in the NIS who were at least 24 months of age at household interview; children aged 19–23 months at interview are excluded from this measure. This commonly used measure for 24 month coverage is statistically inefficient; information about children aged 19–23 months at time of survey is gathered but not used.

#### 24 Month Imputed Coverage

For this third measure of 4:3:1:3:3 coverage, we modeled 24-month coverage for children aged 19–23 months at the time of interview and used previously calculated 24 month coverage for children who were 24–35 months at the time of interview. Modeled immunizations were multiply imputed using 10 imputations. We divided the children into classes based on age at interview and vaccine doses received by age 19 months. Some children's immunization coverage at age 24 months could be determined either retrospectively (aged 24 months or more at time of interview) or prospectively (aged 19–23 months and: had all doses so will still have them at age 24 months; or had so few doses at interview that being fully immunized at age 24 months was implausible). The 24 month immunization status of some children aged 19–23 months at interview was uncertain. For these children, we constructed a logistic regression model based on demographics. See Appendix 1 (Additional file 1 and Table 2)for details.

### Statistical Methods

We compared 19–35 month coverage and 24 month coverage, obtained by both methods, nationally, by race/ethnicity, by poverty status, and by state for both years of NIS data. Percentages are reported with 95% confidence intervals. All analyses were conducted using SAS release 8.02 and SAS-callable SUDAAN release 8.0.0, a software package designed for the analysis of complex survey data.

## Results

Table 1 presents 4:3:1:3:3 vaccination coverage estimates, using the three different measures, nationally and by race/ethnicity, poverty status, and state (only 2002 results are presented; 2001 results were similar). The percent of imputed cases (unweighted) also appears in Table 1.

**Table 1:** Comparison of estimated 4:3:1:3:3* vaccination coverage among children using three different measures, United States, National Immunization Survey, Q1/2002-Q4/2002†

**Level of analysis (percent of children aged 19–23 months at time of household interview, unweighted)**	**Estimates for 4:3:1:3:3 Coverage (95% Confidence Interval)**		
	
	**19–35 Month **(n = 21,410)	**24 Month (Complete case) **(n = 14,910)	**24 Month (Imputed)‡ **(n = 21,410)
**Overall **(30.4%)	74.8 (73.8, 75.8)	72.9 (71.7, 74.1)	72.7 (71.6, 73.8)

**Race/Ethnicity:**			
Hispanic (31.3%)	72.8 (70.4, 75.1)	72.4 (69.6, 75.2)	71.1 (68.6, 73.6)
White, non-Hispanic (29.7%)	77.5 (76.4, 78.8)	75.3 (73.9, 76.7)	75.2 (74.0, 76.5)
Black, non-Hispanic (31.6%)	67.5 (64.6, 70.5)	63.8 (60.0, 67.5)	65.1 (61.8, 68.3)
All others, non-Hispanic (31.0%)	75.4 (70.8, 80.0)	74.4 (69.3, 79.5)	74.1 (69.3, 78.9)

**Poverty Status:**			
Above poverty (30.2%)	77.0 (75.9, 78.2)	74.7 (73.3, 76.1)	74.9 (73.6, 76.2)
Near poverty (28.4%)	67.6 (62.4, 72.8)	68.2 (62.7, 73.6)	65.9 (60.6, 71.2)
Intermediate poverty (31.7%)	70.4 (67.1, 73.8)	68.1 (64.0, 72.2)	68.1 (64.6, 71.6)
Severe poverty (31.2%)	67.7 (64.1, 71.4)	68.1 (63.8, 72.3)	66.4 (62.5, 70.3)

**State:**			
Alabama (33.3%)	76.8 (71.5, 82.1)	72.6 (65.6, 79.6)	73.6 (67.9, 79.3)
Alaska (31.3%)	75.3 (69.4, 81.2)	67.8 (60.2, 75.4)	68.1 (61.4, 74.8)
Arizona (28.7%)	67.9 (63.2, 72.7)	68.8 (63.3, 74.4)	66.9 (61.7, 72.0)
Arkansas (27.3%)	71.0 (64.9, 77.1)	69.9 (62.9, 76.9)	70.5 (64.0, 76.9)
California (33.4%)	73.2 (69.4, 77.0)	73.4 (68.9, 77.9)	72.3 (68.2, 76.5)
Colorado (26.3%)	62.8 (56.1, 69.4)	60.1 (52.3, 67.9)	58.7 (51.5, 66.0)
Connecticut (27.3%)	81.9 (76.7, 87.1)	77.9 (71.6, 84.2)	78.3 (72.8, 83.8)
Delaware (31.3%)	78.7 (73.2, 84.2)	78.2 (71.6, 84.8)	77.7 (71.7, 83.7)
District of Columbia (27.4%)	69.7 (62.2, 77.2)	66.7 (58.2, 75.2)	67.3 (59.2, 75.5)
Florida (30.2%)	74.5 (69.8, 79.2)	71.3 (65.4, 77.2)	71.1 (65.7, 76.4)
Georgia (32.8%)	80.4 (76.1, 84.6)	80.3 (75.4, 85.2)	78.7 (74.3, 83.1)
Hawaii (34.6%)	78.7 (73.2, 84.3)	73.4 (66.0, 80.8)	75.8 (69.6, 82.0)
Idaho (31.0%)	69.4 (63.4, 75.3)	66.6 (59.1, 74.0)	67.5 (61.2, 73.8)
Illinois (30.1%)	78.6 (74.3, 82.9)	76.8 (71.4, 82.1)	77.2 (72.7, 81.7)
Indiana (29.8%)	76.0 (71.0, 81.0)	78.3 (72.7, 84.0)	75.1 (69.0, 81.2)
Iowa (31.5%)	78.7 (73.2, 84.2)	77.5 (71.2, 83.9)	77.1 (71.3, 82.8)
Kansas (32.0%)	66.8 (59.9, 73.7)	66.5 (58.5, 74.6)	65.8 (58.8, 72.9)
Kentucky (29.8%)	72.3 (65.9, 78.7)	67.5 (59.6, 75.5)	69.6 (62.5, 76.7)
Louisiana (29.6%)	66.8 (61.2, 72.5)	66.4 (60.1, 72.7)	66.3 (60.3, 72.3)
Maine (29.8%)	80.7 (75.6, 85.8)	76.9 (70.3, 83.5)	76.8 (71.1, 82.5)
Maryland (34.4%)	78.7 (73.1, 84.4)	76.3 (69.7, 82.9)	76.5 (70.4, 82.7)
Massachusetts (28.5%)	86.2 (82.5, 90.0)	81.8 (76.4, 87.1)	82.8 (78.5, 87.1)
Michigan (29.9%)	81.6 (77.2, 86.0)	75.8 (69.5, 82.0)	77.0 (71.8, 82.3)
Minnesota (27.7%)	76.8 (70.3, 83.4)	78.5 (71.8, 85.2)	76.1 (69.2, 83.0)
Mississippi (31.1%)	75.7 (69.2, 82.3)	74.4 (65.9, 82.9)	73.3 (65.9, 80.8)
Missouri (28.8%)	73.0 (66.5, 79.6)	66.9 (58.8, 75.0)	69.2 (62.4, 76.1)
Montana (29.7%)	66.6 (59.8, 73.5)	62.6 (54.2, 71.1)	63.6 (56.5, 70.7)
Nebraska (24.8%)	78.2 (72.6, 83.8)	77.5 (71.0, 84.0)	75.8 (69.5, 82.1)
Nevada (28.3%)	76.4 (70.3, 82.4)	74.4 (67.2, 81.6)	75.9 (69.5, 82.2)
New Hampshire (28.8%)	83.5 (78.6, 88.5)	78.4 (72.0, 84.8)	80.9 (75.4, 86.4)
New Jersey (30.1%)	76.1 (70.7, 81.6)	73.1 (65.8, 80.4)	73.0 (66.7, 79.3)
New Mexico (30.9%)	64.6 (57.9, 71.4)	61.2 (52.6, 69.8)	61.2 (54.0, 68.4)
New York (27.9%)	77.5 (73.2, 81.8)	77.9 (73.0, 82.9)	76.6 (72.1, 81.1)
North Carolina (32.4%)	82.4 (76.9, 88.0)	80.3 (73.5, 87.2)	79.8 (73.7, 85.9)
North Dakota (27.7%)	77.7 (71.0, 84.4)	73.5 (64.9, 82.1)	74.8 (67.7, 81.9)
Ohio (29.5%)	75.0 (70.5, 79.6)	72.2 (66.9, 77.6)	71.9 (66.9, 76.8)
Oklahoma (33.6%)	65.3 (57.9, 72.7)	63.1 (54.2, 72.0)	63.3 (55.4, 71.1)
Oregon (33.9%)	70.0 (64.1, 75.9)	64.6 (56.9, 72.3)	65.5 (59.1, 71.8)
Pennsylvania (29.6%)	74.7 (69.3, 80.2)	72.7 (65.9, 79.5)	74.7 (69.0, 80.3)
Rhode Island (32.0%)	84.5 (78.9, 90.1)	78.2 (70.7, 85.6)	81.1 (75.2, 86.9)
South Carolina (32.6%)	78.8 (72.3, 85.3)	77.2 (69.2, 85.1)	77.3 (70.4, 84.1)
South Dakota (33.7%)	79.9 (73.5, 86.3)	77.2 (68.7, 85.7)	75.8 (68.6, 82.9)
Tennessee (31.1%)	78.2 (74.1, 82.3)	75.8 (70.7, 80.9)	76.1 (71.5, 80.7)
Texas (28.6%)	67.9 (62.8, 72.9)	65.2 (59.2, 71.2)	65.3 (60.1, 70.5)
Utah (28.5%)	75.7 (69.9, 81.6)	73.8 (66.8, 80.7)	74.8 (68.6, 81.1)
Vermont (30.6%)	80.9 (76.1, 85.6)	75.2 (69.0, 81.5)	75.4 (69.9, 80.9)
Virginia (31.3%)	72.0 (65.8, 78.2)	68.6 (60.8, 76.3)	70.5 (63.9, 77.1)
Washington (29.6%)	69.2 (64.2, 74.2)	64.8 (59.0, 70.7)	65.1 (59.9, 70.3)
West Virginia (32.4%)	76.9 (70.6, 83.1)	75.8 (69.0, 82.6)	74.2 (67.4, 80.9)
Wisconsin (30.6%)	80.3 (75.9, 84.6)	79.6 (75.1, 84.1)	78.1 (73.6, 82.6)
Wyoming (30.6%)	73.3 (67.0, 79.7)	72.0 (64.4, 79.6)	70.5 (64.0, 77.1)

* 4:3:1:3:3 Four or more doses of any diphtheria and tetanus toxoids and pertussis vaccines including diphtheria and tetanus toxoids, and any acellular pertussis vaccine (DTP/DTaP/DT), three or more doses of poliovirus vaccine, one or more doses of any measles containing vaccine (MCV), three or more doses of Haemophilus influenzae type b (Hib), and three or more doses of hepatitis B vaccine. † Children in the Q1/2002-Q4/2002 National Immunization Survey were born between February 1999 and June 2001; n = 21,410. ‡ 10 imputations were performed.

**Table 2:** Logistic regression model used for the multiple imputation analysis

Variable	Odds for intercept, odds ratio otherwise	95% confidence interval
Intercept	0.06	0.03 – 0.10
Race: Hispanic	0.79	0.61 – 1.03
White, non-Hispanic	REF	REF
Black, non-Hispanic	0.73	0.53 – 0.99
All others, non-Hispanic	0.95	0.61 – 1.46
Census Region: Northeast	1.90	1.36 – 2.65
Midwest	1.42	1.04 – 1.94
South	1.28	0.97 – 1.69
West	REF	REF
MSA*: MSA, central city	REF	REF
MSA, non-central city	1.20	0.95 – 1.51
Non-MSA	0.91	0.68 – 1.23
Number of DTP^† ^doses at 19 months: 4+ doses^‡^	2.94	2.37 – 3.65
3 doses	REF	REF
Number of MCV^§ ^doses at 19 months: 1+ doses^||^	2.82	2.11 – 3.80
0 doses	REF	REF
Number of hepatitis B doses at 19 months: 3+ doses^¶^	3.86	2.82 – 5.28
2 doses	0	0

* Metropolitan Statistical Area (MSA)

† Diphtheria and tetanus toxoids, and any acellular pertussis vaccine (DTP)

‡ Four or more doses (4+)

§Measles-containing vaccine (MCV)

|| One or more doses (1+)

¶Three or more doses (3+)

**Figure 1:**
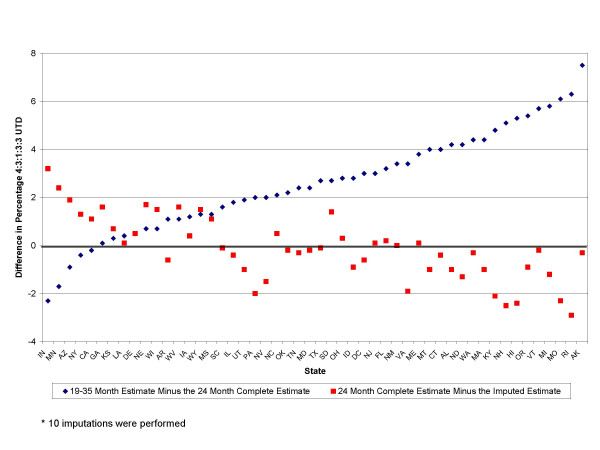
Difference Between the 19–35 month, 24 month complete-case, and the 24-month multiple-imputation* estimates, National Immunization Survey, 2002.

At the national level, the difference between the 19–35 month estimate and the 24 month complete-case estimate was 1.9 percentage points. For most states and subgroups, the 19–35 month estimate was higher than the 24 month complete-case estimate. This can also be seen in Figure 1, which displays the difference between the coverage measures by state. The difference between vaccination coverage measured at 19–35 months and 24 months ranged from -2.3 to 7.5 percentage points among states. For three states, the difference between the 19–35 month and 24 month complete-case estimate was more than 6 percentage points, in twelve states there was a 4–6 percentage point difference, and in sixteen states a 2–4 percentage point difference. Conversely, five states had higher 24 month complete-case estimates than 19–35 month estimates; these were: Indiana, Minnesota, Arizona, New York, and California (Table 1 and Figure 1). Two income subgroups, those near poverty and those in severe poverty, also had higher 24 month complete-case coverage than 19–35 month coverage (Table 1).

Comparing the 24 month complete-case estimates to the 24 month imputed estimates, nationally the imputed measure was 0.2 percentage points lower than the complete-case estimate. The imputed estimates were about equally divided between being greater than or less than the 24 month complete-case estimates (Table 1). As expected, the 24 month imputed estimates had, with the exception of two states (Indiana and Minnesota), narrower confidence intervals than the 24 month complete case estimates. The authors found no explanation of why this was so; in 2001 (results not presented), only one state (North Carolina) had a complete case confidence interval that was narrower than the imputed confidence interval.

The percentage decrease in standard error from using the multiple imputation measure of 4:3:1:3:3 coverage over using the complete case estimate was 8% ([SE complete case-SE imputed case]/ [SE complete case]= [0.61–0.56]/ [0.61]= 0.08). When the imputation model was applied to those for which 24 month coverage was known, the immunization status was correctly assigned for approximately 60%. Note that random variation among imputations was added as an additional component to the model.

## Discussion

The problem of determining immunization coverage at age 24 months is well-suited to multiple imputation. Data are missing at random. The NIS telephone interviews are conducted on a national basis throughout the year, making the age of child at time of telephone interview independent of demographics. Age of child at household interview alone determines if a child's immunization coverage at age 24 months is missing.

In comparing coverages, we found that, at the national level, 19–35 month 4:3:1:3:3 coverage was slightly higher than both the 24 month complete-case and imputed estimates (Table 1). However, at the state level there were much larger differences. For five states the 24 month coverage estimates were larger than the 19–35 month estimates. For 2001 (data not shown), 11 states, all different from those of 2002, had larger 24 month coverage than 19–35 month coverage. The authors found no common characteristics of these states; perhaps larger 24 month coverage is simply random variation, and is not explainable by state characteristics.

How can 24 month coverage estimates be larger than 19–35 month coverage? Approximately 29% (percent varies slightly among years) of the children in the NIS are younger than 24 months at time of household interview. In the 24 month complete-case estimate, these children are excluded from both numerator and denominator. If, because of lower incidence of delayed vaccination or increased incidence of very delayed vaccination (age 24–35 months) or by chance, coverage among 24–35 month old children was substantially lower than among those aged 19–23 months, the estimate for 19–35 month olds would be lower than coverage measured at 24 months of age.

We found that our multiple imputation resulted in slightly narrower confidence intervals for all except two states. One could narrow the confidence intervals for the complete case analysis by increasing the sample size. If we can assume that the hypothetical increased sample size would not change the design effect, then to achieve on a national scale the same precision from the complete case analysis that multiple imputation yielded would require a sample size 1.19 times as large ([width of complete case confidence interval/width of imputed case confidence interval]^2^). To increase the NIS sample size by this magnitude would require an addition of approximately $2.6 million to the survey budget. The improvement in precision is more substantial for some states; as an arbitrarily chosen example, we would need a sample 1.51 times as large in Alabama to get the same improvement in precision that we get from imputation by increasing sample size. Thus, use of multiple imputation produces an essentially cost-free increase in precision of the 24 month vaccination coverage estimates that would be costly and difficult to equal through increased sample size alone. Additionally, because the data are not missing completely at random, but are missing at random, generally there is bias in complete-case methods that is mostly removed (depending on the quality of the imputation model) by multiple imputation.

The potential precision gains to be achieved must be considered in light of drawbacks inherent in this approach. One potential drawback of using multiple imputation is that a predictive model must be specified. However, a body of both analytic and simulation-based work supports the claim that the model used in multiple imputation can be quite far off and still result in substantially better answers than other methods; thus the importance of the "multiple" part of multiple imputation.[24] In this study our model performed only modestly in classifying UTD status of those for whom we knew UTD status; vaccination behaviors involve a complex, and not fully understood interaction of personal beliefs, attitudes, and knowledge, of health provider variables, and system variables.

## Conclusion

Many published results concern vaccination coverage among 19–35 month old children [8,9,22]. This is a moving cohort, consisting of children whose age is between 19 and 35 months at any point during a given calendar year; many children in one year's cohort are also in the next year's. Coverage for 19–35 month old children reflects vaccination status at time of household interview; a child's age at time of interview impacts coverage, with older children more likely to be vaccinated. In short, coverage among 19–35 month old children is difficult to interpret while coverage at age 24 months is readily interpreted, and is a common standard [10-18].

Can one directly compare 19–35 month old coverage with 24 month coverage? We have shown that 19–35 month old coverage can be either smaller or greater than 24 month coverage. Thus, neither coverage measure bounds the other. We have shown that coverage at 19–35 months and at 24 months can, at best, serve as crude surrogates for one another, particularly at a state level. We would, for most purposes, caution against direct comparison (although, for those who wish to make direct comparison, our methods provide a measure of the likely differences between these immunization coverages).

We have shown that multiple imputation can yield more precise estimates of coverage at 24 months than is obtained by a complete case analysis, at essentially no increase in cost. Similar methods are applicable to other public health surveys. With today's limited resources for public health surveys, we recommend that multiple imputation be used, where appropriate, to improve precision.

## Competing interests

The author(s) declare that they have no competing interests.

## Authors' Contributions

All authors participated in the analyses and drafted the manuscript. All authors read and approved the final manuscript.

## Supplementary Material

Additional File 1 Appendix 1. Click here for file
