# Supplementary material for: Measurement of vaccination coverage at age 24 and 19–35 months: a case study of multiple imputation in public health
Source: Popul Health Metr. 2005 Jul 5;3:6. doi: 10.1186/1478-7954-3-6 (PMC1190214; doi:10.1186/1478-7954-3-6)
Supplement: Additional File 1 — Appendix 1. [file 1478-7954-3-6-S1.doc]

**APPENDIX 1**

In multiple imputation, a model is constructed that describes the distribution of the missing data, given the available data. Multiple simulated data sets are created; in each, missing data are randomly generated from the fitted model. Point estimates are obtained by averaging the random data sets’ point estimates. The variance of the resulting point estimate is the sum of the between (how different are the random data sets from one another?) and within (how much sampling variability does the point estimate have for a single random data set?) realization variances.[2]

To perform the multiple imputation, we divided children in the 2002 NIS into six mutually exclusive subsets:

A. Interviewed at 19-23 months; 4:3:1:3:3 UTD at 19 months

B. Interviewed at 19-23 months; not 4:3:1:3:3 UTD at 19 months; very few doses and thus unlikely to become UTD by 24 months

C. Interviewed at 19-23 months; not 4:3:1:3:3 UTD at 19 months; not in class B

D. Interviewed at 24-35 months; 4:3:1:3:3 UTD at 19 months

E. Interviewed at 24-35 months; not 4:3:1:3:3 UTD at 19 months; very few doses

F. Interviewed at 24-35 months; not 4:3:1:3:3 UTD at 19 months; not in class E

Children in class A will be 4:3:1:3:3 UTD at 24 months because they were UTD at 19 months, thus we coded them as being UTD at 24 months. Children in class B were unlikely to become UTD at 24 months due to receipt of so few doses by 19 months of age (< 2 doses of DTaP, < 1 dose of polio, < 1 dose of hib, or < 1 dose of hep B); they are therefore coded as not UTD at 24 months. We made this assumption because only 4 out of 1361 children in group E went on to become UTD at 24 months. For children in classes D, E, and F, no imputation was needed because their coverage at 24 months was known. For children in class C we imputed 24 month 4:3:1:3:3 coverage.

To impute UTD/not UTD coverage for children in class C, we built a logistic regression model using children in class F, those children most similar to those in class C. We performed backward elimination logistic regression with 4:3:1:3:3 UTD status at 24 months as the outcome variable. The explanatory variables initially entered into the model included: number of children in the household, first born status, income/poverty level, race/ethnicity of the child, census region, Metropolitan Statistical Area (MSA), gender, mother’s education level, age and marital status, number of DTP vaccine doses by 19 months of age, number of poliovirus vaccine doses by 19 months of age, number of MCV doses by 19 months of age, number of Hib vaccine doses by 19 months of age, and number of hepatitis B vaccine doses by 19 months of age. At each step we eliminated from the model the variable having the highest *P*-value until all factors had *P ≤* 0.15. The final model included as explanatory variables: child’s race, census region, MSA, number of DTP vaccine doses by 19 months of age, number of MCV doses by 19 months of age, and number of hepatitis B vaccine doses by 19 months of age. This was the final model from which we obtained parameter estimates and the variance-covariance matrix for the next step of the imputation.

Once we had constructed the logistic regression model, we generated ten sets of vectors from a multivariate normal distribution having as mean the vector of parameters from the final multiple logistic regression model and variance-covariance matrix the variance-covariance matrix of the model parameters.

For each multiple imputation realization for the children in class C, we calculated the logit of 4:3:1:3:3 UTD status at 24-month by multiplying one of the ten parameter vectors from the multivariate normal distribution by the design matrix. We then transformed this variable by exp(x)/(1+exp(x)) to obtain the modeled probability of being UTD. To create a dichotomous variable defining 4:3:1:3:3 UTD status, we utilized a uniform[0,1] random variable generated for each child. If the predicted probability was less than the random uniform variable then the child was considered not UTD; if the predicted probability was greater than or equal to the random uniform variable then the child was considered UTD; this was the 4:3:1:3:3 imputed UTD status.[2] We then calculated 4:3:1:3:3 UTD coverage estimates for each of ten multiple imputation realizations on all children using the multiple imputation model for class C combined with the UTD status as previously determined for classes A, B, D, E, and F. The mean of the 10 imputed estimates is the multiple imputation estimate. The standard error of this estimate was computed taking into account both within and between imputation variances.

We performed 10 realizations for the multiple-imputation. This is an arbitrary, but reasonable, number. For multiple imputations, degrees of freedom is determined by the within replication and between replication variance. For this problem, ten realizations resulted in the number of degrees of freedom being greater than 1000. This indicates that additional realizations would provide little additional information. Table 2 displays the resulting odds (odds ratios) and 95% confidence interval from the logistic regression model used for the multiple imputation analyses. (The variance-covariance matrix of these 18 parameter estimates is available from the authors upon request.) Table 2 shows that, as one might expect, immunization status at age 19 months is much more important in determining immunization status at age 24 months than are demographics. The multiple imputation method utilized in this paper is an attempt to create a good model, but it is just one choice among many possibilities.
